# Differential methylation of *SNCA* and *MAPT* genes associated with Parkinson’s disease in Mexican Mestizos

**DOI:** 10.3389/fnagi.2025.1612544

**Published:** 2025-07-09

**Authors:** Ernesto G. Miranda-Morales, Daniel F. Ramos-Rosales, Alma C. Salas-Leal, Sergio M. Salas-Pacheco, Francisco X. Castellanos-Juárez, Edna M. Méndez-Hernández, Osmel La Llave-León, María S. Peñaherrera, Gerardo Quiñones-Canales, Oscar Arias-Carrión, Ada A. Sandoval-Carrillo, José M. Salas-Pacheco

**Affiliations:** ^1^Instituto de Investigación Científica, Universidad Juárez del Estado de Durango, Durango, Mexico; ^2^Facultad de Odontología, Universidad Juárez del Estado de Durango, Durango, Mexico; ^3^Department of Medical Genetics, University of British Columbia, Vancouver, BC, Canada; ^4^Hospital General Santiago Ramón y Cajal-ISSSTE, Durango, Mexico; ^5^División de Neurociencias | Clínica, Instituto Nacional de Rehabilitación Luis Guillermo Ibarra Ibarra, Mexico City, Mexico; ^6^Unidad de Trastornos del Movimiento y Sueno, Hospital General Dr. Manuel Gea González, Mexico City, Mexico

**Keywords:** Parkinson’s disease, DNA methylation, *SNCA*, *MAPT*, Mexican Mestizos

## Abstract

**Introduction:**

Parkinson’s disease (PD) is a neurodegenerative disorder characterized by motor symptoms such as bradykinesia, rigidity, and tremor. Despite its prevalence, genetic and epigenetic studies in the Mexican Mestizo population (individuals of mixed Indigenous and European—primarily Spanish—ancestry who represent the majority demographic in Mexico) remain limited. DNA methylation may play a role in PD pathogenesis, with peripheral blood methylation patterns serving as potential biomarkers. This study examines *MAPT* and *SNCA* gene methylation in Mexican PD patients to identify epigenetic alterations associated with the disease.

**Methods:**

In this case-control study, we enrolled 108 PD patients and 108 age- and sex matched controls from Mexico City and Durango. Genomic DNA was extracted from leukocytes, and bisulfite pyrosequencing was performed to assess methylation levels at specific CpG sites within *MAPT* and *SNCA*.

**Results:**

Our analysis revealed a significant reduction in global methylation levels in the *MAPT* promoter region and *SNCA* intron 1 in PD patients compared to controls (*MAPT*, *p* = 0.0019; *SNCA*, *p* = 0.000069). Site-specific analysis showed significant hypomethylation at *MAPT* CpG sites 1, 4, 10–11 and at *SNCA* CpG sites 1–3, 5–7 and, 25 in PD cases. Regional analysis (central and northern México) revealed significant differences in *MAPT* methylation between PD patients and controls exclusively in the northern region (*p* = 0.0039) and in *SNCA* methylation only in the central region (*p* = 0.00001). Gender-based stratification indicated that *MAPT* methylation was significantly different in men (*p* = 0.0013), whereas *SNCA* methylation differences were significant only in women (*p* = 0.00045). We found an association between global methylation patterns of *MAPT* (OR = 1.182, 95% CI = 1.029–1.197, *p* = 0.007) and *SNCA* (OR = 1.243, 95% CI = 1.067–1.448, *p* = 0.005) with PD. Gender-stratified regression showed that *MAPT* methylation was significantly associated with PD exclusively in men (OR = 1.182, 95% CI = 1.041–1.342, *p* = 0.010), whereas *SNCA* methylation was significantly associated with PD only in women (OR = 1.337, 95% CI = 1.044–1.713, *p* = 0.021).

**Conclusion:**

Our findings reveal significant *MAPT* and *SNCA* hypomethylation in PD patients within the Mexican Mestizo population. These epigenetic modifications may contribute to PD pathogenesis and highlight the potential of DNA methylation profiles as biomarkers for PD, particularly in regional- and gender-specific contexts. This study advances the understanding of PD’s molecular mechanisms and underscores the importance of studying diverse populations to identify novel disease biomarkers.

## 1 Introduction

Parkinson’s disease (PD) is a progressive neurodegenerative disorder affecting approximately 1% of individuals over 60 years of age, characterized by tremor, bradykinesia, and rigidity, all of which significantly impact patients’ quality of life (Balestrino and Schapira, 2020). In Mexico, the prevalence of PD is estimated at 40 to 50 cases per 100,000 individuals annually, contributing to the global burden of an estimated 6.1 million affected individuals (Velázquez-Paniagua et al., 2016; Tysnes and Storstein, 2017; GBD 2016 Parkinson’s Disease Collaborators, 2018). Despite advancements in understanding PD’s motor symptoms, effective curative or neuroprotective treatments remain elusive. Genetic research has identified mutations and polymorphisms in several PD-associated genes, underscoring their role as susceptibility factors. However, the genetic and biochemical mechanisms underlying PD in the Mexican population remain largely unexplored (Salas-Leal et al., 2019; Romero-Gutierrez et al., 2021; Salas-Leal et al., 2021; Salas-Leal et al., 2023). The underrepresentation of Latin American populations in neurogenomic research has hindered the identification of ancestry-specific epigenetic signatures relevant to disease susceptibility. In this context, the Mexican Mestizo population (the predominant demographic group in Mexico, composed of individuals with mixed Indigenous and primarily Spanish European ancestry) provides a genetically distinct landscape shaped by historical admixture, offering an opportunity to uncover novel epigenetic mechanisms underlying PD pathogenesis (Martínez-Cortés et al., 2012; Silva-Zolezzi et al., 2009).

The *MAPT* and *SNCA* genes have been implicated in PD pathogenesis. *MAPT* encodes the microtubule-associated protein tau, which plays a key role in microtubule stabilization and axonal transport. Its misfolding and aggregation have been observed in PD, suggesting a multifaceted role in neuronal dysfunction (Zhang Y. et al., 2018; Zhang X. et al., 2018). In contrast, *SNCA* encodes α-synuclein, whose abnormal aggregation is a key event for Lewy body formation, a hallmark of PD pathology (Pascale et al., 2016; Goedert et al., 2017; Pedersen et al., 2021).

Epigenetic modifications, particularly DNA methylation changes, have been associated with various neurological disorders, including PD (Urdinguio et al., 2009; Klein and De Jager, 2016). Recent studies on DNA methylation patterns in brain and peripheral blood samples of PD patients have revealed concordant epigenetic landscapes, suggesting that peripheral blood may serve as a viable surrogate for brain tissue in PD epigenetic research (Masliah et al., 2013; Pihlstrom et al., 2015). These insights highlight the potential of epigenetic markers in PD diagnosis and pathogenesis, particularly through non-invasive approaches.

Given the global prevalence of PD and the genetic diversity of the Mexican population, our study aims to characterize the methylation profiles of *MAPT* and *SNCA* in leukocytes of Mexican PD patients. This research seeks to bridge the existing knowledge gap in PD’s genetic and epigenetic underpinnings within this demographic, offering new insights into disease mechanisms and potential biomarkers for PD.

## 2 Materials and methods

### 2.1 Subjects

This case-control study encompassed 108 Parkinson’s disease (PD) cases and 108 age- and sex-matched controls, devoid of any apparent neurological disorders. Participants were recruited from the Movement Disorders Unit at Dr. Manuel Gea González General Hospital, the Neurology Service of General Hospital 450, and Santiago Ramón y Cajal Hospital at ISSSTE in Durango.

Inclusion criteria for all participants included being between 50 and 85 years of age, self-reported Mexican Mestizo ancestry (defined as having at least three generations of ancestors born in Mexico), and willingness to provide written informed consent. In addition, PD patients were required to have a clinical diagnosis of idiopathic Parkinson’s disease according to the United Kingdom Parkinson’s Disease Society Brain Bank criteria (Gibb and Lees, 1988) and a minimum disease duration of 1 year, while healthy controls had to be free of neurological or psychiatric disorders and have no family history of PD. Exclusion criteria for both groups comprised the presence of atypical or secondary parkinsonism (e.g., drug-induced or vascular), other neurodegenerative or psychiatric disorders, active inflammatory or autoimmune diseases, recent infections, or use of immunosuppressive or anti-inflammatory medications within 3 months prior to sample collection.

**Figure 1:**
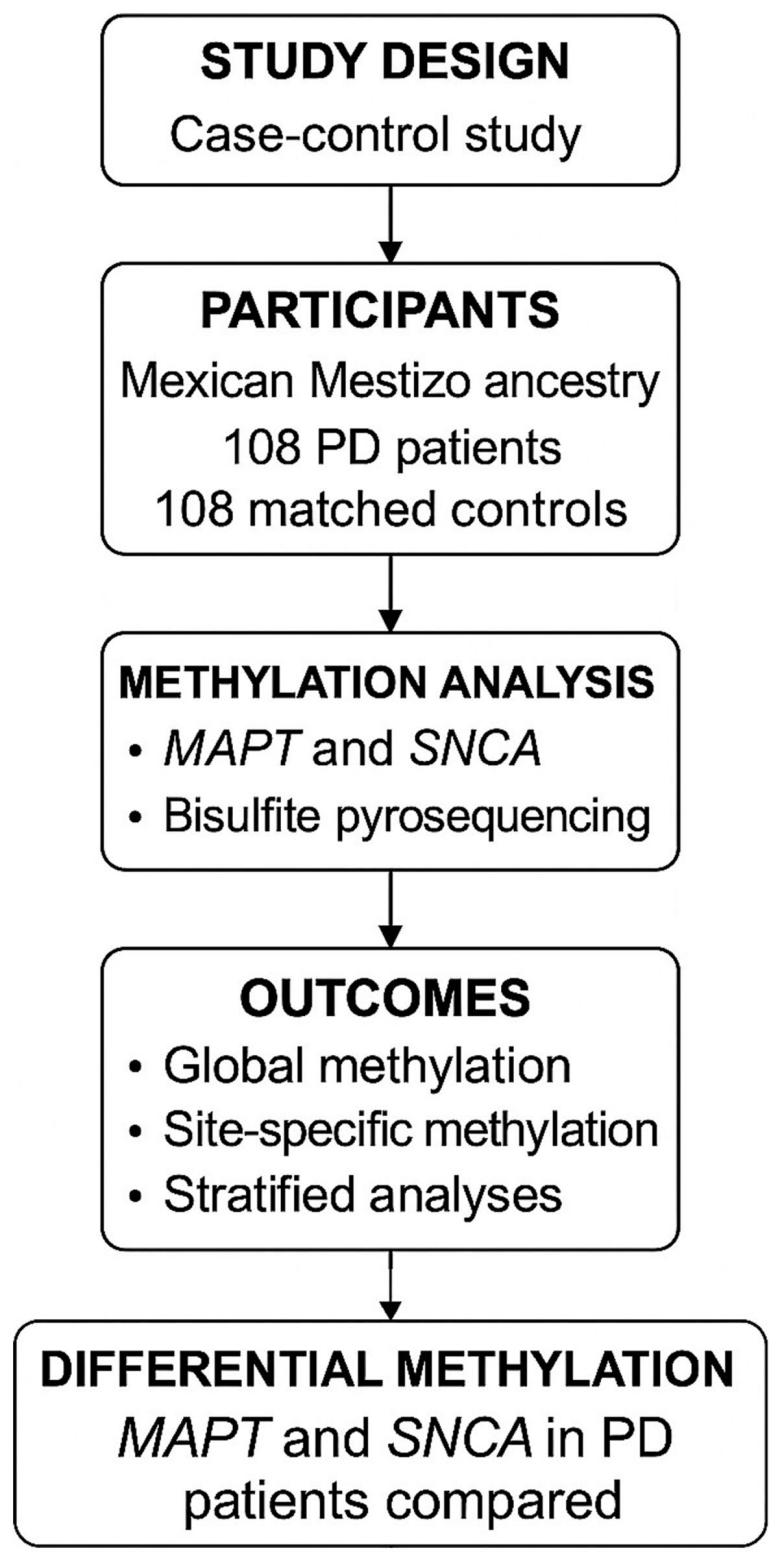
Study design and methodological workflow.

The study was conducted in accordance with the Declaration of Helsinki and was approved by the Research Ethics Committees of all participating institutions (ISSSTE Hospital General Dr. Santiago Ramon y Cajal: EeI/056/13; Hospital General Dr. Manuel Gea González: CI y CEI/111/15; Hospital General 450 Durango: 23/08/12). Written informed consent was obtained from all participants prior to enrollment. A visual summary of the study design and methodological workflow is provided in Figure 1.

### 2.2 Clinical assessment

PD cases underwent comprehensive evaluations using the Movement Disorder Society-modified Unified Parkinson’s Disease Rating Scale (UPDRS), Mini-Mental State Examination (MMSE), and Hamilton Rating Scale for Depression (HDRS) to assess motor function, cognitive status, and depression severity, respectively. Controls were assessed for cognitive function and depression using the MMSE and HDRS.

### 2.3 DNA isolation

To perform DNA extraction from blood samples, the protocol described by Iranpur and Esmailizadeh (2010) was employed. The qualitative assessment of DNA was performed on a 1% agarose gel stained with ethidium bromide. The quantitative assessment was performed using 1 μL of the sample through spectrophotometry at a wavelength of 260 and 280 nm on a Nanodrop 2000 spectrophotometer.

### 2.4 Methylation profile assessment

The analysis of methylation patterns was performed using the pyrosequencing method following the protocol described by Hogg et al. (2013). Specific probes from the EpiTect Methyl II PCR primer catalog were used for each region under evaluation. For *MAPT* CpGs 1–11: AGTAAGGAGAAAGGAAGTAGTT (forward), ACTAAATCCCCTACCCTTTACTTTCAAT (reverse), and CTACCCTTTACTTTCAATC (sequencing). For *SNCA* CpGs 1–7: AGAGAGGGTAGATAGATTTTATGTGAGA (forward), AATAAATACTTATCCCTTTAAAAAACCT (reverse), and AGAGAAAAGTTGGATGT (sequencing). For *SNCA* CpG25: GATGGTGGAAAGGAG TAGGTAATAGAA (forward), CCAACTTTTCTCTCACATAAAATCTATCTA (reverse), and GTGGAAAGGAGT AGGTAATAGAAT (sequencing). Bisulfite conversion was performed using the EZ DNA Methylation Gold Kit (Zymo Research) following the manufacturer’s protocol. Pyrosequencing was carried out using the Pyromark Q96 MD P instrument (Qiagen Ltd.).

### 2.5 Statistical analysis

Descriptive statistics were utilized to summarize the data. The student’s *t*-test (or Mann–Whitney *U* test for non-normally distributed data) was applied to compare quantitative variables between groups. The chi-square test (or Fisher’s exact test for small sample sizes) assessed differences in categorical variables. Normality of data distribution was verified using Kolmogorov–Smirnov and Shapiro–Wilk tests. Linear regression analysis explored the relationships between variables. All statistical analyses were performed using SPSS v.21.0 (IBM Corp.), and a two-tailed *p*-value <0.05 was considered statistically significant.

### 2.6 Ethical considerations

The study received approval from the Ethics Committees of all participating institutions. Informed consent was obtained from all participants, ensuring voluntary participation and confidentiality in line with ethical guidelines for human research.

## 3 Results

### 3.1 Study population characteristics

Our study cohort consisted of 216 participants, including 108 PD patients and 108 age- and sex-matched controls, recruited from both central (Mexico City) and northern (Durango) regions of Mexico. Detailed clinical and sociodemographic characteristics are summarized in Table 1. Comparative analysis revealed significant differences between PD patients and controls in body mass index (BMI; *p* = 0.009), serum uric acid levels (*p* = 0.010), depression severity assessed by the Hamilton scale (*p* = 0.034), leukocyte counts (*p* = 0.009), mean corpuscular hemoglobin (MCH; *p* = 0.002), and mean corpuscular hemoglobin concentration (MCHC; *p* < 0.001).

**Table 1:** Clinical and sociodemographic characteristics of the study groups.

Variable	Cases PD (*n* = 108)	Controls (*n* = 108)	*p*
Age, years	70.23 ± 9.16	69.97 ± 9.20	0.836^a^
Males *n* (%)/Females *n* (%)	56 (51.8)/52 (48.2)	54 (50)/54 (50)	0.785^c^
North *n* (%)/Center *n* (%)	69 (63.9)/39 (36.1)	69 (63.9)/39 (36.1)	1.000^c^
BMI, kg/m^2^	26.86 ± 6.07	23.33 ± 10.92	0.009^a^
Glucose, mg/dL	97.1/ (80.0–117.9)	93.2 (86.8–106.9)	0.323^b^
Total cholesterol, mg/dL	183.58 ± 51.75	193.88 ± 36.01	0.095^a^
Uric acid, mg/dL	5.35 ± 2.30	6.03 ± 1.31	0.010^a^
Creatinine, mg/dL	0.94 ± 0.55	1.04 ± 0.30	0.209^a^
MMSE, points	25.12 ± 4.61	25.56 ± 3.21	0.475^a^
HDRS, points	11.40 ± 7.42	9.26 ± 5.15	0.034^a^
Leukocytes, (×10^3^/μL)	5.9 (4.35–6.90)	6.5 (5.4–8.2)	0.009^b^
Erythrocytes, (×10^6^/μL)	4.76 (4.45–5.05)		0.287^b^
Hemoglobin, g/dL	14.4 (13.35–15.65)	13.9 (13.00–15.50)	0.216^b^
HCT, %	42.3 (39.20–47.20)	43.3 (39.70–46.50)	0.706^b^
MCV, fL	89.9 (84.70–96.20)	91.70 (88.00–94.10)	0.305^b^
MCH, pg	31.1 (29.55–32.65)	29.6 (28.50–30.9)	0.002^b^
MCHC, g/dL	34.6 (33.10–35.80)	31.9 (31.00–33.50)	<0.001^b^
Platelets, (×10^3^/μL)	203.0 (141.0–276.0)	222.0 (181.0–260.0)	0.315^b^

HCT, hematocrit; MCV, mean corpuscular volume; MCH, mean corpuscular hemoglobin; MCHC, mean corpuscular hemoglobin concentration.

a Student’s *t*.

b Mann–Whitney *U*.

c *χ*^2^.

### 3.2 Methylation levels in *MAPT* and *SNCA* genes

**Figure 2:**
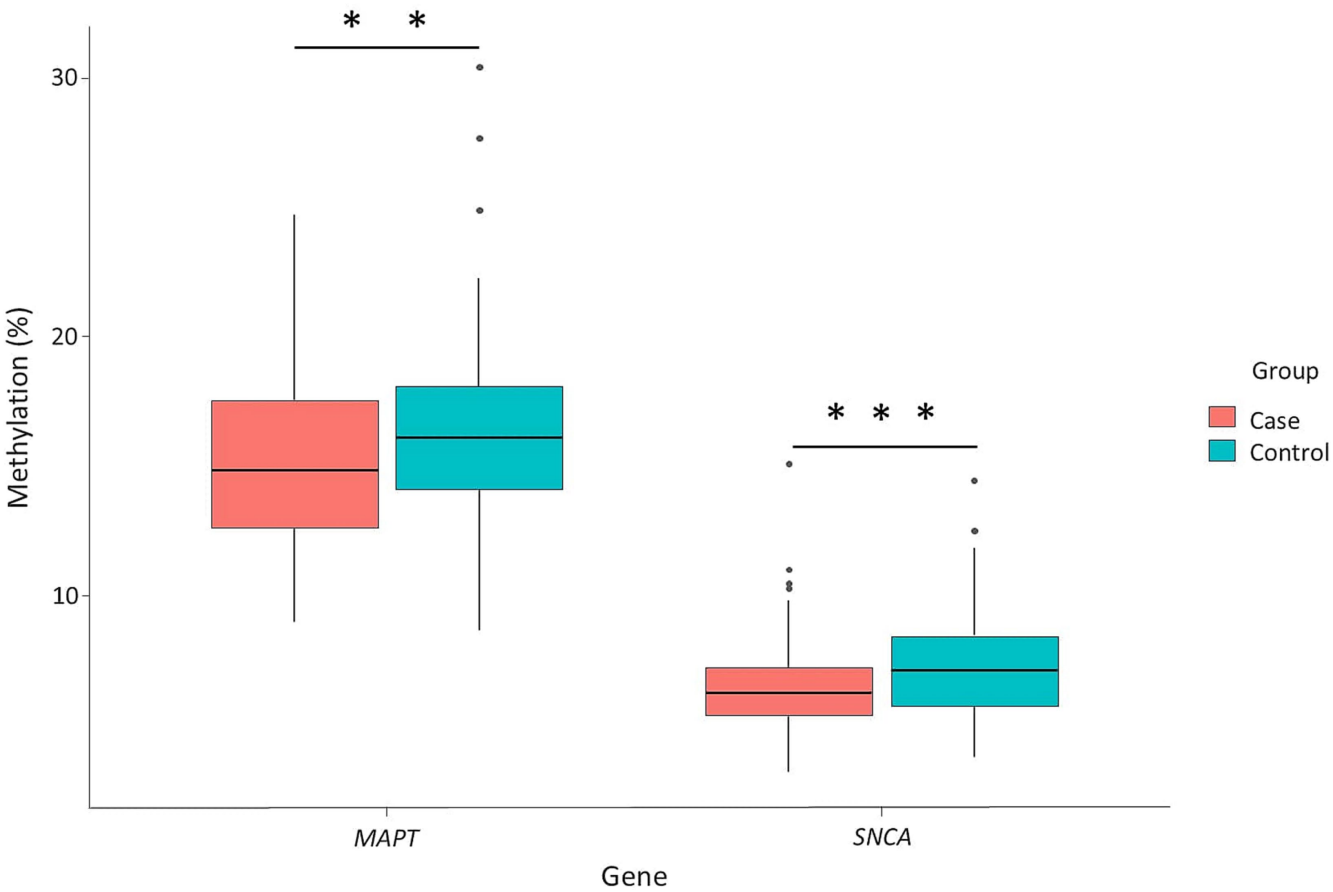
Differential global methylation levels in *MAPT* and *SNCA* genes between PD patients and controls. Box plots display the median (horizontal line inside the box) and the 25th and 75th percentiles (box borders). Outliers are denoted by closed circles. ^**^p < 0.01, and ^***^p < 0.001.

Analysis of global methylation patterns within the *MAPT* gene promoter region (CpGs 1–11) and the *SNCA* gene intron 1 (CpGs 1–7 and CpG 25) revealed significant hypomethylation in the PD group compared to controls (*MAPT*, *p* = 0.0019; *SNCA*, *p* = 0.000069), as illustrated in Figure 2.

**Figure 3:**
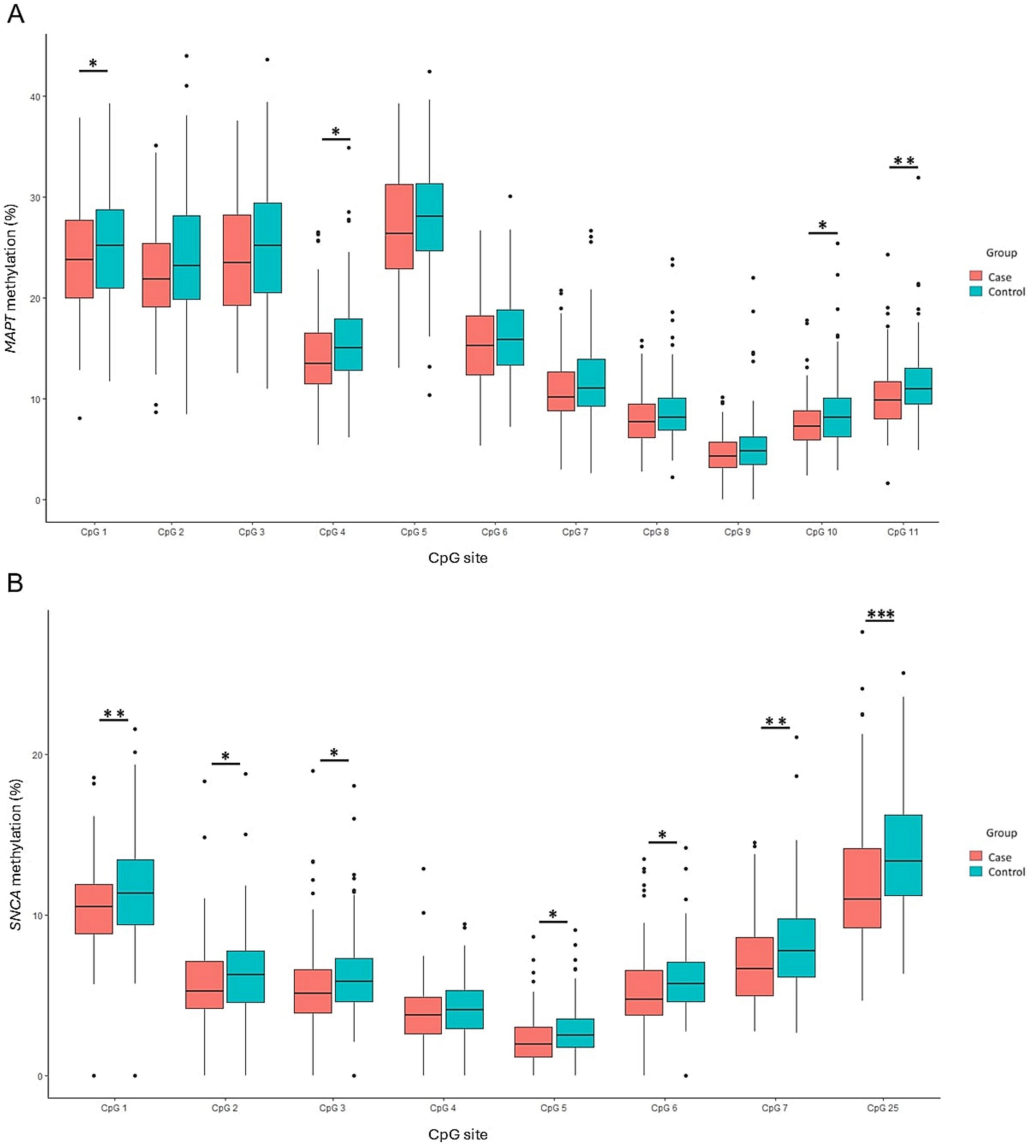
Comparison of methylation patterns at each CpG site in the *MAPT* **(A)** and *SNCA* **(B)** genes between PD patients and controls. Box plots display the median (horizontal line inside the box) and the 25th and 75th percentiles (box borders). Outliers are denoted by closed circles. ^*^p < 0.05, ^**^p < 0.01, and ^***^p < 0.001.

Decreased methylation levels were observed across all examined CpG sites in the *MAPT* gene among PD patients, with significant differences at CpGs 1, 4, 10, and 11 (*p* = 0.04, *p* = 0.014, *p* = 0.018, and *p* = 0.004, respectively) (Figure 3A). Similarly, in the *SNCA* gene, all the specific CpG sites exhibited reduced methylation in PD patients, with significant differences at CpGs 1, 2, 3, 5, 6, 7, and 25 (*p* = 0.007, *p* = 0.04, *p* = 0.018, *p* = 0.016, *p* = 0.012, *p* = 0.003, and *p* = 0.0002 respectively) (Figure 3B).

### 3.3 Regional stratification of methylation patterns

**Figure 4:**
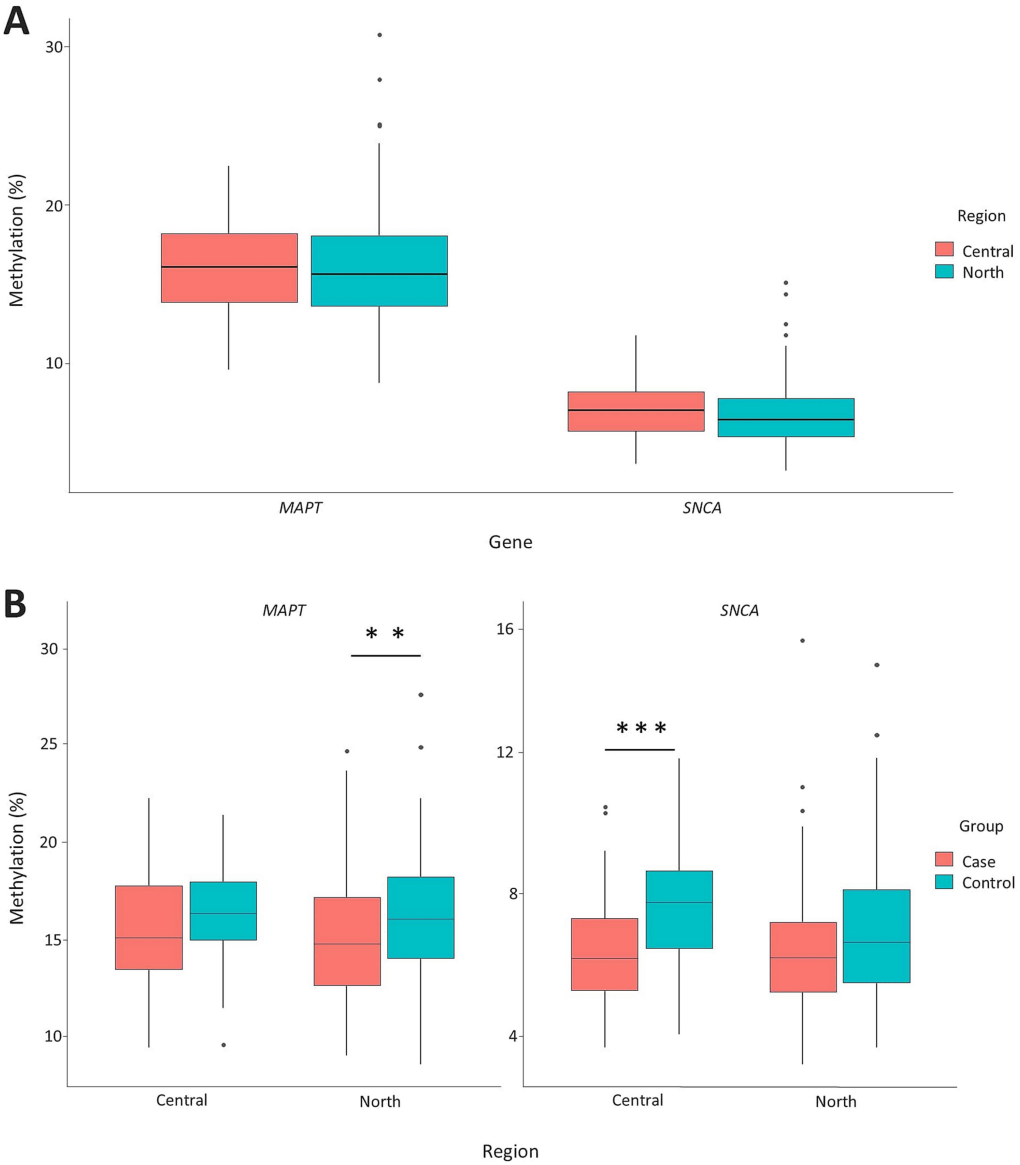
Regional comparison of *MAPT* and *SNCA* gene methylation patterns. Overview of methylation differences in the *SNCA* and *MAPT* genes across central and north regions within the study population **(A)**. Comparison of *MAPT* and *SNCA* gene methylation levels between PD patients and controls, stratified by region **(B)**. Box plots display the median (horizontal line inside the box) and the 25th and 75th percentiles (box borders). Outliers are denoted by closed circles. ^*^p < 0.05 and ^**^p < 0.01.

**Figure 5:**
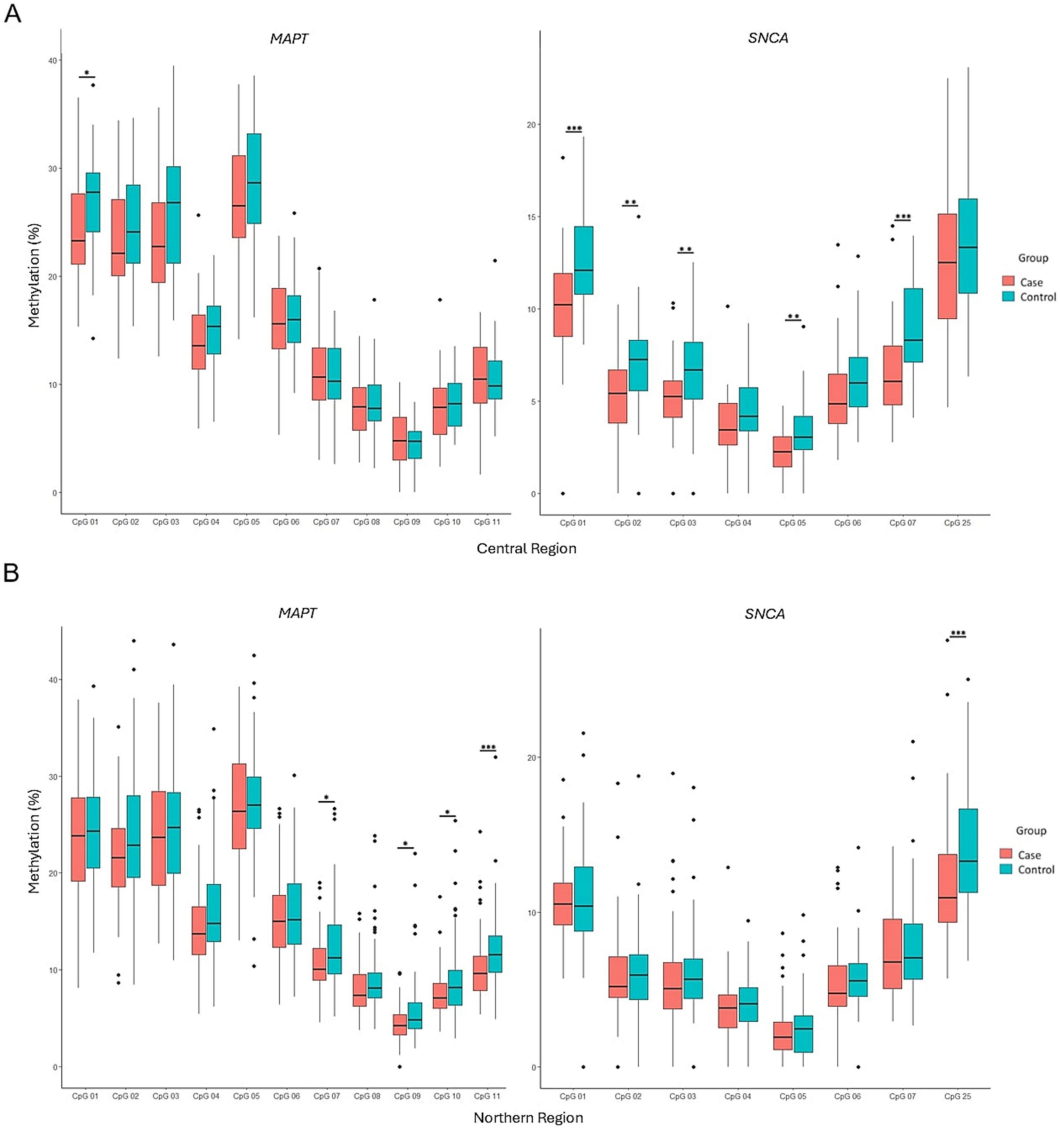
Comparison of methylation patterns at each CpG site in the *MAPT* and *SNCA* genes between PD patients and controls in the central **(A)** and northern **(B)** regions. Box plots display the median (horizontal line inside the box) and the 25th and 75th percentiles (box borders). Outliers are denoted by closed circles. ^*^p < 0.05, ^**^p < 0.01, and ^***^p < 0.001.

Regional analysis of participants from Mexico City (central) and Durango (northern) showed no significant differences in global methylation levels of the *MAPT* and *SNCA* genes between the two regions (Figure 4A). However, stratified comparisons revealed significant differences in *MAPT* methylation in the northern region (*p* = 0.0039) and *SNCA* methylation in the central region (*p* = 0.00001) between PD patients and controls (Figure 4B). In the central region, significant differences in *SNCA* methylation between PD patients and controls were observed at CpG sites 1, 2, 3, 5, and 7 (*p* = 0.0002, *p* = 0.0075, *p* = 0.0042, *p* = 0.0052, and *p* = 0.00013, respectively), in contrast, *MAPT* showed differential methylation only at CpG site 1 (*p* = 0.0306) (Figure 5A). In the northern region, *MAPT* methylation was significantly different at CpG sites 7, 9, 10, and 11 (*p* = 0.0184, *p* = 0.0168, *p* = 0.0423, and *p* = 0.00034, respectively), whereas *SNCA* exhibited differential methylation only at CpG site 25 (*p* = 0.000283) (Figure 5B).

### 3.4 Gender-based stratification of methylation patterns

**Figure 6:**
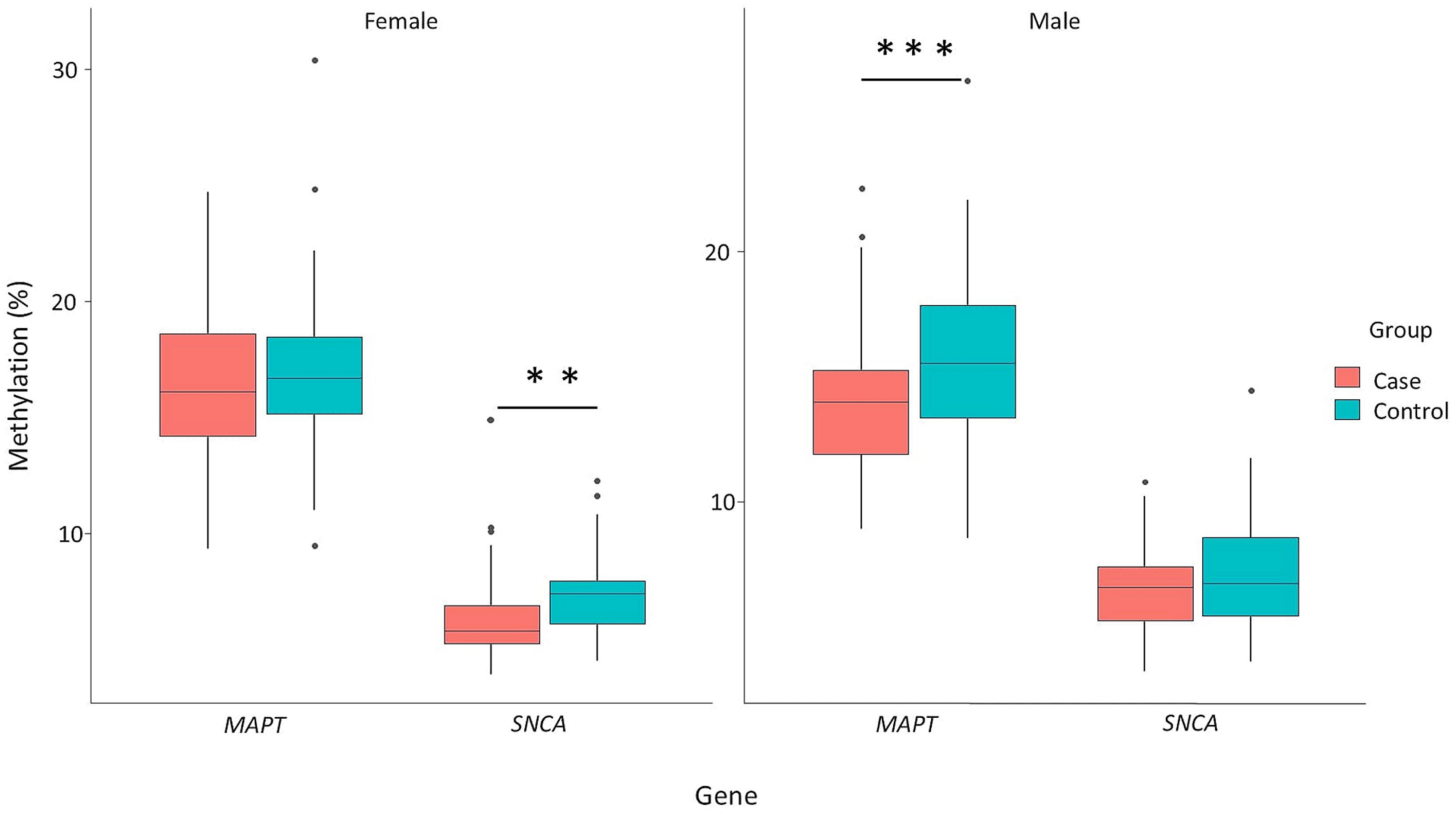
Comparison of *MAPT* and *SNCA* genes methylation patterns stratified by gender. The box plots show the median (horizontal line inside the box) and the 25th and 75th percentiles (horizontal borders of the box). Outliers are denoted by closed circles. ^**^p < 0.01 and ^***^p < 0.001.

**Figure 7:**
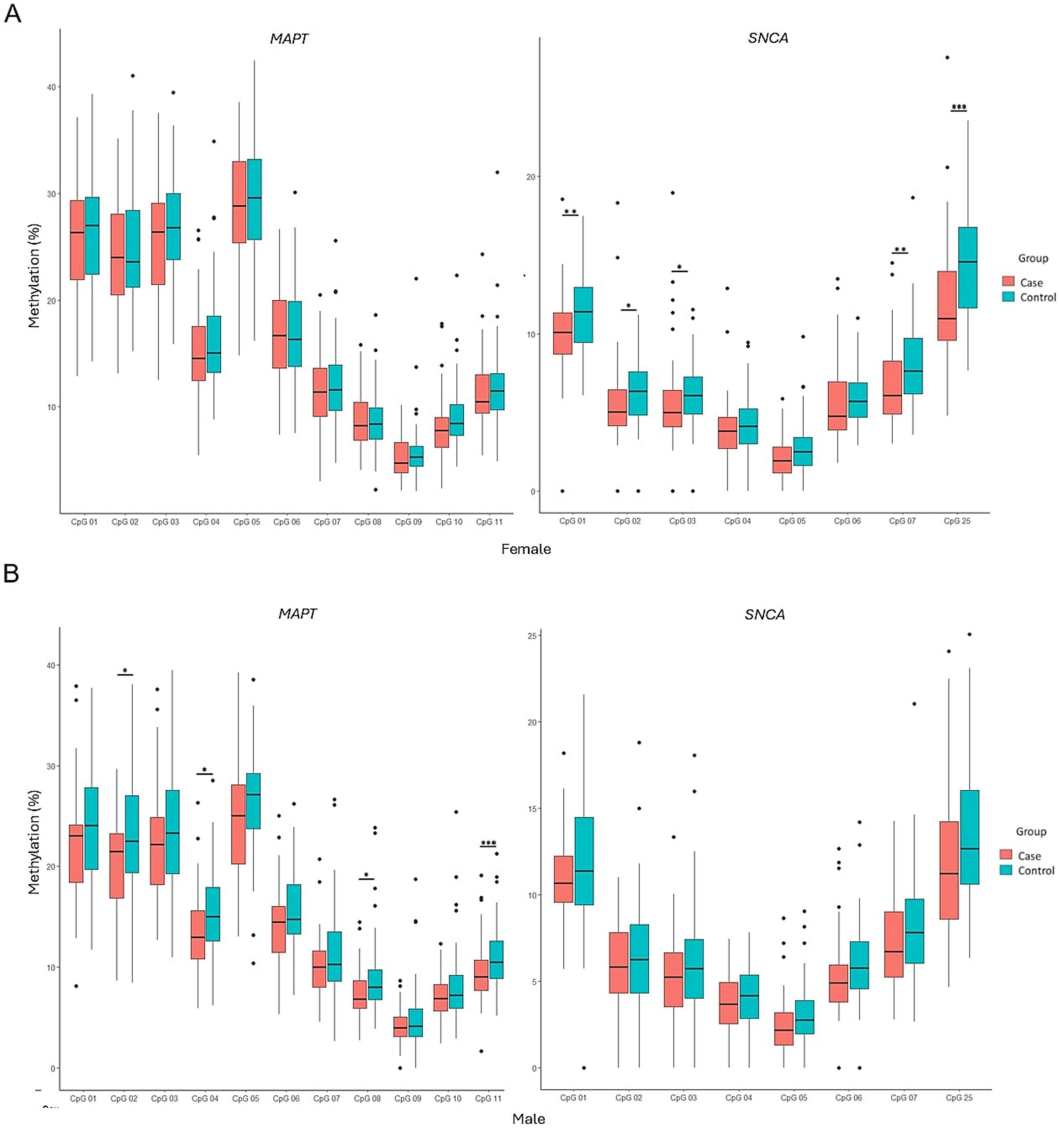
Comparison of methylation patterns at each CpG site in the *MAPT* and *SNCA* genes between PD patients and controls in females **(A)** and males **(B)**. Box plots display the median (horizontal line inside the box) and the 25th and 75th percentiles (box borders). Outliers are denoted by closed circles. ^*^p < 0.05, ^**^p < 0.01, and ^***^p < 0.001.

The influence of gender on *MAPT* and *SNCA* gene methylation was analyzed. The results indicate that significant differences in *MAPT* methylation were observed only in men (*p* = 0.0013), whereas significant differences in *SNCA* methylation were exclusive to women (*p* = 0.00045). Hypomethylation of both *MAPT* and *SNCA* remained a prominent feature in PD patients (Figure 6). CpG site-specific comparisons showed that, within the male subgroup, significant differences between PD patients and controls were observed at CpG2, CpG4, CpG8, and CpG11 of the *MAPT* gene (*p* = 0.0223, *p* = 0.0185, *p* = 0.0346, and *p* = 0.00882, respectively) (Figure 7A). Conversely, in the female subgroup, significant differences were detected at CpG1, CpG2, CpG3, CpG6, CpG7, and CpG25 of the *SNCA* gene (Figure 7B).

### 3.5 Association of methylation profile with Parkinson’s disease

Binary logistic regression analyses demonstrate an association between global methylation patterns of the *MAPT* (OR = 1.182, 95% CI = 1.029–1.197, *p* = 0.007) and *SNCA* (OR = 1.243, 95% CI = 1.067–1.448, *p* = 0.005) genes and PD. Gender-stratified regression further revealed that *MAPT* methylation was significantly associated with PD exclusively in men (OR = 1.182, 95% CI = 1.041–1.342, *p* = 0.010). In contrast, *SNCA* methylation was significantly associated with PD only in women (OR = 1.337, 95% CI = 1.044–1.713, *p* = 0.021).

## 4 Discussion

Our study contributes to the growing body of evidence supporting the role of epigenetic mechanisms, particularly DNA methylation, in the pathogenesis of PD. By focusing on the Mexican Mestizo population, which remains largely underrepresented in epigenetic and genomic studies, we address a significant gap in current research that has been predominantly centered on European and Asian cohorts. Recent multi-ancestry genome-wide association studies have emphasized the need for ethnic diversity in PD epigenetic research, as the genetic architecture and epigenomic regulation of risk loci like *MAPT* and *SNCA* differ markedly across populations (Kim et al., 2024; Park et al., 2023; Zhang Y. et al., 2018; Zhang X. et al., 2018).

The significant differences observed in BMI, uric acid levels, Hamilton scale scores, leukocyte counts, MCH, and MCHC between PD patients and controls underscore the multifactorial nature of the disease. Our findings on uric acid levels align with global research, including the meta-analysis by Wen et al. (2017), which reinforces the antioxidant and neuroprotective properties of uric acid, as well as its role in modulating iron accumulation—a well-established contributor to dopaminergic neuron degeneration (Waring, 2002; Berg and Hochstrasser, 2006; Schlesinger and Schlesinger, 2008; Sian-Hulsmann et al., 2011; Dusek et al., 2016; Wang et al., 2017; Liu et al., 2019).

The association between PD and alterations in hematological parameters, including leukocyte counts and hemoglobin content, may reflect underlying inflammatory processes and oxidative stress that contribute to neuronal loss (Lin et al., 2014; Freed and Chakrabarti, 2016). These observations suggest that systemic changes in PD extend beyond the central nervous system, offering potential biomarkers for disease detection and monitoring.

Our analysis of *MAPT* and *SNCA* methylation revealed a distinct pattern of hypomethylation in PD patients, consistent with previous studies linking epigenetic modifications to disease pathogenesis (Pihlstrom et al., 2015; Schmitt et al., 2015). Masliah et al. (2013) provided direct evidence that concordant DNA methylation changes can be observed in both brain and blood samples from PD patients, reinforcing the utility of peripheral tissues for biomarker discovery and mechanistic inference.

The observed hypomethylation across *MAPT* CpGs sites, particularly CpGs 1, 4, 10, and 11, adds new insight to previously mixed findings, underscoring the complex interplay between tau protein dysregulation and PD. While Coupland et al. (2014) reported differential methylation of *MAPT* across brain regions and peripheral blood, they did not assess the specific CpG sites examined in our study. Furthermore, Masliah et al. (2013) demonstrated concordant methylation changes between brain and blood in PD, but did not investigate the *MAPT* locus. Thus, our findings provide, to our knowledge, the first site-specific evidence of *MAPT* hypomethylation in peripheral blood of PD patients. These discrepancies support the notion of tissue- and ancestry-specific dynamics in *MAPT* epigenetic regulation. Notably, these epigenetic changes were identified in the Mexican Mestizo population, highlighting the possibility that epigenetic marks may vary in frequency or regulatory function across diverse genetic backgrounds (Romero-Gutierrez et al., 2021).

Similarly, the hypomethylation at *SNCA* CpGs sites 1, 2, 3, 5, 6, and 7, including CpG25, a previously unexplored site, suggests a nuanced regulatory mechanism affecting α-synuclein levels. These CpG sites are located within intron 1 and, according to the GRCh38/hg38 annotation, are predicted to reside within a transcriptional control region. Notably, CpG25 exhibited the most pronounced difference between PD patients and controls, indicating its potential role in *SNCA* gene regulation. Our findings are consistent with previous reports of intron 1 hypomethylation in peripheral blood mononuclear cells from PD patients (Guhathakurta et al., 2017), as well as in cerebellum, putamen, and frontal cortex from *post-mortem* brain tissue (Jowaed et al., 2010). More recently, Gu et al. (2021) demonstrated that this hypomethylation is specific to neuronal cells. These epigenetic alterations align with functional studies indicating that reduced methylation at intron 1 increases *SNCA* expression, and have been associated with earlier onset and more rapid progression of PD (Fedotova et al., 2023; Jowaed et al., 2010).

Geographic stratification revealed that *MAPT* hypomethylation was more pronounced among patients from northern Mexico, whereas *SNCA* hypomethylation was more significant in those from central Mexico, indicating potential regional influences on epigenetic regulation. These differences may be driven by environmental or lifestyle factors (such as exposure to pesticides or air pollutants, both recognized contributors to PD) that could affect DNA methylation patterns in a region-specific manner (Dorsey and Bloem, 2024; Nandipati and Litvan, 2016). Supporting this hypothesis, a study using a mouse model chronically exposed to MPTP (1-methyl-4-phenyl-1,2,3,6-tetrahydropyridine), a prototypical neurotoxin used to model PD, reported altered methylation at intron 1 of SNCA in brain tissue, providing mechanistic evidence for toxin-induced epigenetic modulation of this gene (Schaffner and Kobor, 2022).

Additionally, our findings indicate a gender-specific pattern of hypomethylation: *MAPT* was significantly hypomethylated in men, while *SNCA* hypomethylation was predominant in women. This may contribute to sex-based differences in PD susceptibility and progression. In men, *MAPT* hypomethylation likely enhances its expression, which may disrupt cytoskeletal integrity and accelerate neurodegeneration. Overexpression of *MAPT* and other PD-related genes has been associated with increased cellular energy demands, particularly in mitochondrial function and calcium homeostasis (Simunovic et al., 2010). This supports the hypothesis that elevated metabolic activity in dopaminergic neurons may accelerate neuronal aging and increase PD risk (Flores-Ponce and Velasco, 2024). Hypomethylation of intron 1 in *SNCA* has been linked to increased gene expression in *post-mortem* brain tissue from patients with sporadic PD (Ai et al., 2014), potentially leading to alpha-synuclein accumulation and heightened neuronal vulnerability in women. In *SNCA*-knockout mice, reduced bone loss following ovariectomy suggests that this protein may interact with hormonal processes (Lisofsky et al., 2015). Additionally, women with PD are reported to experience tremors more frequently and exhibit more rapid nigrostriatal degeneration than men (Haaxma et al., 2007), which may be linked to differential *SNCA* expression in dopaminergic neurons of the substantia nigra compacta and potentially modulated by estrogen activity.

Several limitations should be considered when interpreting our findings. This study focused exclusively on two candidate genes (*MAPT* and *SNCA*), limiting the ability to capture broader epigenomic alterations associated with PD. Although significant methylation differences were observed, the lack of functional validation (such as gene expression analysis or mechanistic assays) precludes conclusions about their biological relevance. The cross-sectional design further limits causal inference, as methylation changes may reflect disease progression or treatment effects rather than etiological mechanisms. Moreover, analyses were conducted on peripheral blood leukocytes without adjustment for potential cellular heterogeneity, and other confounding factors such as age, medication use, or environmental exposures were not accounted for. The absence of correction for multiple testing across CpG sites increases the risk of false positives. Lastly, findings were derived from a single, region-specific cohort without replication in an independent sample, which may limit the generalizability of the results to other populations.

## 5 Conclusion

This study reveals significant hypomethylation in the *MAPT* and *SNCA* genes in peripheral blood leukocytes of Mexican Mestizo patients with Parkinson’s disease. These epigenetic alterations are associated with the disease in a region- and sex-specific manner, highlighting the potential utility of DNA methylation profiles as accessible, non-invasive biomarkers for PD.

Our findings support the growing recognition that epigenetic mechanisms contribute to neurodegeneration and underscore the relevance of population-specific studies. The observed hypomethylation patterns, particularly in *MAPT* among men and *SNCA* among women, offer novel insights into the sex-dependent epigenetic regulation of PD risk.

Future investigations are warranted to validate the functional consequences of the observed methylation changes through gene expression and protein assays, thereby confirming their biological relevance. Longitudinal studies are essential to elucidate how these epigenetic patterns evolve throughout disease progression. Moreover, expanding analyses to brain tissue or neural cell models could clarify their relevance to central neuropathology. Finally, genome-wide approaches and replication in diverse populations will be critical to identify broader and more generalizable epigenetic signatures.

## Data Availability

The data supporting the findings of this study have been deposited in Figshare and are publicly available at the following DOI: https://doi.org/10.6084/m9.figshare.29441972.

## References

[ref1] Ai S. X. Xu Q. Hu Y. C. Song C. Y. Guo J. F. Shen L.. (2014). Hypomethylation of SNCA in blood of patients with sporadic Parkinson’s disease. J. Neurol. Sci. 337, 123–128. doi: 10.1016/j.jns.2013.11.033, PMID: 24326201

[ref2] Balestrino R. Schapira A. H. V. (2020). Parkinson disease. Eur. J. Neurol. 27, 27–42. doi: 10.1111/ene.14108, PMID: 31631455

[ref3] Berg D. Hochstrasser H. (2006). Iron metabolism in parkinsonian syndromes. Mov. Disord. 21, 1299–1310. doi: 10.1002/mds.21020, PMID: 16817199

[ref4] Coupland K. G. Mellick G. D. Silburn P. A. Mather K. Armstrong N. J. Sachdev P. S.. (2014). DNA methylation of the MAPT gene in Parkinson’s disease cohorts and modulation by vitamin E *in vitro*. Mov. Disord. 29, 1606–1614. doi: 10.1002/mds.25784, PMID: 24375821 PMC4074263

[ref5] Dorsey E. R. Bloem B. R. (2024). Parkinson’s disease is predominantly an environmental disease. J. Parkinsons Dis. 14, 451–465. doi: 10.3233/JPD-230357, PMID: 38217613 PMC11091623

[ref6] Dusek P. Schneider S. A. Aaseth J. (2016). Iron chelation in the treatment of neurodegenerative diseases. J. Trace Elem. Med. Biol. 38, 81–92. doi: 10.1016/j.jtemb.2016.03.010, PMID: 27033472

[ref7] Fedotova E. Y. Iakovenko E. V. Abramycheva N. Y. Illarioshkin S. N. (2023). SNCA gene methylation in Parkinson’s disease and multiple system atrophy. Epigenomes 7:5. doi: 10.3390/epigenomes7010005, PMID: 36810559 PMC9944792

[ref8] Flores-Ponce X. Velasco I. (2024). Dopaminergic neuron metabolism: relevance for understanding Parkinson’s disease. Metabolomics 20:116. doi: 10.1007/s11306-024-02181-4, PMID: 39397188 PMC11471710

[ref9] Freed J. Chakrabarti L. (2016). Defining a role for hemoglobin in Parkinson’s disease. npj Parkinsons Dis. 2:16021. doi: 10.1038/npjparkd.2016.21, PMID: 28725702 PMC5516577

[ref10] GBD 2016 Parkinson’s Disease Collaborators (2018). Global, regional, and national burden of Parkinson’s disease, 1990–2016: a systematic analysis for the Global Burden of Disease Study 2016. Lancet Neurol. 17, 939–953. doi: 10.1016/S1474-4422(18)30295-3, PMID: 30287051 PMC6191528

[ref11] Gibb W. R. Lees A. J. (1988). The relevance of the Lewy body to the pathogenesis of idiopathic Parkinson’s disease. J. Neurol. Neurosurg. Psychiatry 51, 745–752. doi: 10.1136/jnnp.51.6.745, PMID: 2841426 PMC1033142

[ref12] Goedert M. Jakes R. Spillantini M. G. (2017). The synucleinopathies: twenty years on. J. Parkinsons Dis. 7, S51–S69. doi: 10.3233/JPD-179005, PMID: 28282814 PMC5345650

[ref13] Gu J. Barrera J. Yun Y. Murphy S. K. Beach T. G. Woltjer R. L.. (2021). Cell-type specific changes in DNA methylation of SNCA intron 1 in synucleinopathy brains. Front. Neurosci. 15:652226. doi: 10.3389/fnins.2021.652226, PMID: 33994928 PMC8113398

[ref14] Guhathakurta S. Evangelista B. A. Ghosh S. Basu S. Kim Y. S. (2017). Hypomethylation of intron1 of α-synuclein gene does not correlate with Parkinson’s disease. Mol. Brain 10:6. doi: 10.1186/s13041-017-0285-z, PMID: 28173842 PMC5297217

[ref15] Haaxma C. A. Bloem B. R. Borm G. F. Oyen W. J. Leenders K. L. Eshuis S.. (2007). Gender differences in Parkinson’s disease. J. Neurol. Neurosurg. Psychiatry 78, 819–824. doi: 10.1136/jnnp.2006.103788, PMID: 17098842 PMC2117736

[ref16] Hogg K. Blair J. D. McFadden D. E. von Dadelszen P. Robinson W. P. (2013). Early onset pre-eclampsia is associated with altered DNA methylation of cortisol-signalling and steroidogenic genes in the placenta. PLoS One 8:e62969. doi: 10.1371/journal.pone.0062969, PMID: 23667551 PMC3647069

[ref17] Iranpur V. M. Esmailizadeh A. K.. (2010). Rapid extraction of high quality DNA from whole blood stored at 4°C for long period. Available online at: https://www.protocol-online.org/prot/Protocols/Rapid-Extraction-of-High-Quality-DNA-from-Whole-Blood-Stored-at-4-C-for-Long-Period-4175.html (Accessed June 11, 2019).

[ref18] Jowaed A. Schmitt I. Kaut O. Wüllner U. (2010). Methylation regulates alpha-synuclein expression and is decreased in Parkinson's disease patients’ brains. J. Neurosci. 30, 6355–6359. doi: 10.1523/JNEUROSCI.6119-09.2010, PMID: 20445061 PMC6632710

[ref19] Kim J. J. Vitale D. Otani D. V. Lian M. M. Heilbron K. Iwaki H.. (2024). Multi-ancestry genome-wide association meta-analysis of Parkinson’s disease. Nat. Genet. 56, 27–36. doi: 10.1038/s41588-023-01584-8, PMID: 38155330 PMC10786718

[ref20] Klein H. U. De Jager P. L. (2016). Uncovering the role of the methylome in dementia and neurodegeneration. Trends Mol. Med. 22, 687–700. doi: 10.1016/j.molmed.2016.06.008, PMID: 27423266

[ref21] Lin W. C. Tsai N. W. Huang Y. C. Cheng K. Y. Chen H. L. Li S. H.. (2014). Peripheral leukocyte apoptosis in patients with parkinsonism: correlation with clinical characteristics and neuroimaging findings. Biomed. Res. Int. 2014:635923. doi: 10.1155/2014/635923, PMID: 24795890 PMC3984850

[ref22] Lisofsky N. Martensson J. Eckert A. Lindenberger U. Gallinat J. Kuhn S. (2015). Hippocampal volume and functional connectivity changes during the female menstrual cycle. NeuroImage 118, 154–162. doi: 10.1016/j.neuroimage.2015.06.012, PMID: 26057590

[ref23] Liu C. Liang M. C. Soong T. W. (2019). Nitric oxide, iron and neurodegeneration. Front. Neurosci. 13:114. doi: 10.3389/fnins.2019.00114, PMID: 30833886 PMC6388708

[ref24] Martínez-Cortés G. Salazar-Flores J. Fernández-Rodríguez L. G. Rubi-Castellanos R. Rodríguez-Loya C. Velarde-Félix J. S.. (2012). Admixture and population structure in Mexican-Mestizos based on paternal lineages. J. Hum. Genet. 57, 568–574. doi: 10.1038/jhg.2012.67, PMID: 22832385

[ref25] Masliah E. Dumaop W. Galasko D. Desplats P. (2013). Distinctive patterns of DNA methylation associated with Parkinson disease: identification of concordant epigenetic changes in brain and peripheral blood leukocytes. Epigenetics 8, 1030–1038. doi: 10.4161/epi.25865, PMID: 23907097 PMC3891683

[ref26] Nandipati S. Litvan I. (2016). Environmental exposures and Parkinson’s disease. Int. J. Environ. Res. Public Health 13:881. doi: 10.3390/ijerph13090881, PMID: 27598189 PMC5036714

[ref27] Park K. W. Ryu H. S. Shin E. Park Y. Jeon S. R. Kim S. Y.. (2023). Ethnicity- and sex-specific genome wide association study on Parkinson’s disease. npj Parkinsons Dis 9:141. doi: 10.1038/s41531-023-00580-3, PMID: 37805635 PMC10560250

[ref28] Pascale E. Di Battista M. E. Rubino A. Purcaro C. Valente M. Fattapposta F.. (2016). Genetic architecture of MAPT gene region in Parkinson disease subtypes. Front. Cell. Neurosci. 10:96. doi: 10.3389/fncel.2016.00096, PMID: 27147968 PMC4826864

[ref29] Pedersen C. C. Lange J. Forland M. G. G. Macleod A. D. Alves G. Maple-Grodem J. (2021). A systematic review of associations between common SNCA variants and clinical heterogeneity in Parkinson’s disease. npj Parkinsons Dis. 7:54. doi: 10.1038/s41531-021-00196-5, PMID: 34210990 PMC8249472

[ref30] Pihlstrom L. Berge V. Rengmark A. Toft M. (2015). Parkinson’s disease correlates with promoter methylation in the alpha-synuclein gene. Mov. Disord. 30, 577–580. doi: 10.1002/mds.26073 25545759

[ref31] Romero-Gutierrez E. Vazquez-Cardenas P. Moreno-Macias H. Salas-Pacheco J. Tusie-Luna T. Arias-Carrion O. (2021). Differences in MTHFR and LRRK2 variant’s association with sporadic Parkinson’s disease in Mexican Mestizos correlated to native American ancestry. npj Parkinsons Dis. 7:13. doi: 10.1038/s41531-021-00157-y, PMID: 33574311 PMC7878860

[ref32] Salas-Leal A. C. Salas-Pacheco S. M. Gavilan-Ceniceros J. A. P. Castellanos-Juarez F. X. Mendez-Hernandez E. M. La Llave-Leon O.. (2021). Alpha-syn and SNP rs356219 as a potential biomarker in blood for Parkinson’s disease in Mexican Mestizos. Neurosci. Lett. 754:135901. doi: 10.1016/j.neulet.2021.135901, PMID: 33865938

[ref33] Salas-Leal A. C. Salas-Pacheco S. M. Hernandez-Cosain E. I. Velez-Velez L. M. Antuna-Salcido E. I. Castellanos-Juarez F. X.. (2023). Differential expression of PSMC4, SKP1, and HSPA8 in Parkinson's disease: insights from a Mexican Mestizo population. Front. Mol. Neurosci. 16:1298560. doi: 10.3389/fnmol.2023.1298560, PMID: 38115821 PMC10728481

[ref34] Salas-Leal A. C. Sandoval-Carrillo A. Romero-Gutierrez E. Castellanos-Juarez F. X. Mendez-Hernandez E. M. La Llave-Leon O.. (2019). rs3764435 associated with Parkinson’s disease in Mexican Mestizos: case-control study reveals protective effects against disease development and cognitive impairment. Front. Neurol. 10:1066. doi: 10.3389/fneur.2019.01066, PMID: 31649613 PMC6794556

[ref35] Schaffner S. L. Kobor M. S. (2022). DNA methylation as a mediator of genetic and environmental influences on Parkinson’s disease susceptibility: impacts of alpha-synuclein, physical activity, and pesticide exposure on the epigenome. Front. Genet. 13:971298. doi: 10.3389/fgene.2022.971298, PMID: 36061205 PMC9437223

[ref36] Schlesinger I. Schlesinger N. (2008). Uric acid in Parkinson’s disease. Mov. Disord. 23, 1653–1657. doi: 10.1002/mds.22139, PMID: 18618666

[ref37] Schmitt I. Kaut O. Khazneh H. deBoni L. Ahmad A. Berg D.. (2015). L-dopa increases alpha-synuclein DNA methylation in Parkinson’s disease patients in vivo and in vitro. Mov. Disord. 30, 1794–1801. doi: 10.1002/mds.26319 26173746

[ref38] Sian-Hulsmann J. Mandel S. Youdim M. B. Riederer P. (2011). The relevance of iron in the pathogenesis of Parkinson’s disease. J. Neurochem. 118, 939–957. doi: 10.1111/j.1471-4159.2010.07132.x, PMID: 21138437

[ref39] Silva-Zolezzi I. Hidalgo-Miranda A. Estrada-Gil J. Fernández-López J. C. Uribe-Figueroa L. Contreras A.. (2009). Analysis of genomic diversity in Mexican Mestizo populations to develop genomic medicine in Mexico. Proc. Natl. Acad. Sci. U.S.A. 106, 8611–8616. doi: 10.1073/pnas.0903045106, PMID: 19433783 PMC2680428

[ref40] Simunovic F. Yi M. Wang Y. Stephens R. Sonntag K. C. (2010). Evidence for gender-specific transcriptional profiles of nigral dopamine neurons in Parkinson disease. PLoS One 5:e8856. doi: 10.1371/journal.pone.0008856, PMID: 20111594 PMC2810324

[ref41] Tysnes O. B. Storstein A. (2017). Epidemiology of Parkinson’s disease. J. Neural Transm. 124, 901–905. doi: 10.1007/s00702-017-1686-y, PMID: 28150045

[ref42] Urdinguio R. G. Sanchez-Mut J. V. Esteller M. (2009). Epigenetic mechanisms in neurological diseases: genes, syndromes, and therapies. Lancet Neurol. 8, 1056–1072. doi: 10.1016/S1474-4422(09)70262-5, PMID: 19833297

[ref43] Velázquez-Paniagua M. Vázquez-Álvarez A. M. Valverde-Aguilar G. Vergara-Aragón P. (2016). Current treatments in Parkinson's including the proposal of an innovative dopamine microimplant. Rev. Méd. Hosp. Gen. México 79, 79–87. doi: 10.1016/j.hgmx.2015.10.006

[ref44] Wang N. Jin X. Guo D. Tong G. Zhu X. (2017). Iron chelation nanoparticles with delayed saturation as an effective therapy for Parkinson disease. Biomacromolecules 18, 461–474. doi: 10.1021/acs.biomac.6b01547, PMID: 27989126

[ref45] Waring W. S. (2002). Uric acid: an important antioxidant in acute ischaemic stroke. QJM 95, 691–693. doi: 10.1093/qjmed/95.10.691, PMID: 12324642

[ref46] Wen M. Zhou B. Chen Y. H. Ma Z. L. Gou Y. Zhang C. L.. (2017). Serum uric acid levels in patients with Parkinson’s disease: a meta-analysis. PLoS One 12:e0173731. doi: 10.1371/journal.pone.0173731, PMID: 28319195 PMC5358777

[ref47] Zhang X. Gao F. Wang D. Li C. Fu Y. He W.. (2018). Tau pathology in Parkinson’s disease. Front. Neurol. 9:809. doi: 10.3389/fneur.2018.00809, PMID: 30333786 PMC6176019

[ref48] Zhang Y. Shu L. Sun Q. Pan H. Guo J. Tang B. (2018). A comprehensive analysis of the association between SNCA polymorphisms and the risk of Parkinson’s disease. Front. Mol. Neurosci. 11:391. doi: 10.3389/fnmol.2018.00391, PMID: 30410434 PMC6209653

